# Long‐Term Complications of Neglected Double‐J Stent With Renal and Bladder Stone Formation: A Case Report From Sudan

**DOI:** 10.1002/ccr3.70995

**Published:** 2025-09-28

**Authors:** Abubaker Yassin, Osama Mohamed

**Affiliations:** ^1^ Dongola Specialized Hospital Dongola Northern State Sudan

**Keywords:** case reports, cystolithiasis, neglected ureteral stents, pyelolithotomy

## Abstract

Double‐J (DJ) stents are commonly used to maintain ureteral patency after procedures like ureteral stone removal. Ideally, they should be replaced or removed within 6 to 12 weeks to avoid complications such as encrustation and stone formation. However, prolonged retention can lead to severe outcomes. This case report discusses a patient with a neglected DJ stent, which resulted in both renal and bladder stones. A 35‐year‐old Sudanese woman presented with right loin pain and dysuria. She had a DJ stent inserted 4 years ago after ureteroscopy for ureteral stone removal but did not follow up due to insufficient knowledge about the stent. Imaging revealed renal and bladder stones with significant encrustation. Transurethral cystolitholapaxy was performed successfully for the bladder stone. However, attempts to remove the DJ stent endoscopically were unsuccessful due to a large renal pelvic stone attached to the stent. The patient underwent pyelolithotomy to remove the stone together with the retained DJ. One month later, she had no complications and returned to normal activities. The lack of follow‐up and understanding of the DJ stent led to complications. Extended stent retention increases the risk of encrustation and stone formation. Treatment often involves a multiple operations, including pyelolithotomy and endoscopic procedures, especially in resource‐limited settings. This case highlights the need for comprehensive patient education, timely stent removal, and regular follow‐up to prevent devastating complications from neglected DJ stents. It also emphasizes the importance of tailored treatment strategies in resource‐constrained environments.

**Summary**

- Neglected Double‐J stents can lead to serious complications such as encrustation and stone formation in both the kidney and bladder.
- This case highlights the importance of patient education, timely stent removal, and regular follow‐up to prevent morbidity, especially in resource‐limited settings where delayed intervention may result in complex surgical management.

## 1 Introduction

Double‐J (DJ) stents are essential and frequently utilized devices in urology. Typically, the DJ stent must be replaced or removed within 6 weeks to 6 months to prevent issues such as encrustation, stone formation, fractures, and blockages. Unfortunately, in numerous instances, the stent is neglected [1].

It was first introduced to practice in 1967 by Zimskind et al. [2]. Stents help maintain the ureter patent, promote the resolution of edema, and facilitate healing of mucosal injuries. As a result, they are regarded as an effective approach in the postoperative care of patients with conditions such as ureteric calculi, ureteric strictures, retroperitoneal tumors or fibrosis, uretero‐pelvic junction obstruction, or any iatrogenic ureteric injuries.

The likelihood of stone formation increases with an extended period of DJ stent use. It has been reported that the rates of stent encrustation are 9.2%, 47.5%, and 76.3% when the stent remains in place for 6 weeks, 6–12 weeks, and over 12 weeks, respectively [3].

Forgotten DJ stents can lead to serious complications. The educational level of patients and their families, as well as the counseling provided before and after the procedure, could be crucial in minimizing stent‐related issues [1].

Initial efforts to remove encrusted ureteral stents can be difficult. The approach depends on the extent of stone formation and encrustation at both ends of the stent. Various methods have been used to remove encrusted stents, including open surgery, percutaneous nephrolithotomy (PCNL), extracorporeal shockwave lithotripsy (SWL), cystolitholapaxy, ureteroscopic laser lithotripsy, and cystolitholapaxy [4].

## 2 Patient and Observation

### 2.1 Patient Information

A 35‐year‐old Sudanese woman from a rural area presented to the clinic with mild to moderate, dull, aching pain in the right loin, not radiating or shifting, and accompanied by dysuria. She reported frequent episodes of urinary tract infections treated with oral antibiotics. Her past history was significant for a right ureteric stone 4 years ago, which was treated with ureteroscopy and stone fragmentation. She denied using a DJ stent, with no significant medical history.

### 2.2 Clinical Findings

Based on the physical examination, the patient's overall health appeared to be in good condition, and her vital signs were within clinically accepted normal range apart from pallor. Regarding her abdominal examination, no abnormalities were detected in both the right and left costovertebral areas apart from mild suprapubic tenderness.

### 2.3 Timeline of Current Episode

The patient has a medical history of undergoing a ureteroscopy 4 years ago, which was performed due to the presence of a ureteral stone. Following the fragmentation of the stone, a DJ stent was inserted to maintain the patency of the ureter. However, the patient had limited awareness about the DJ stent insertion and did not maintain regular follow‐up after the urological procedure.

### 2.4 Diagnostic Assessment

**FIGURE 1:**
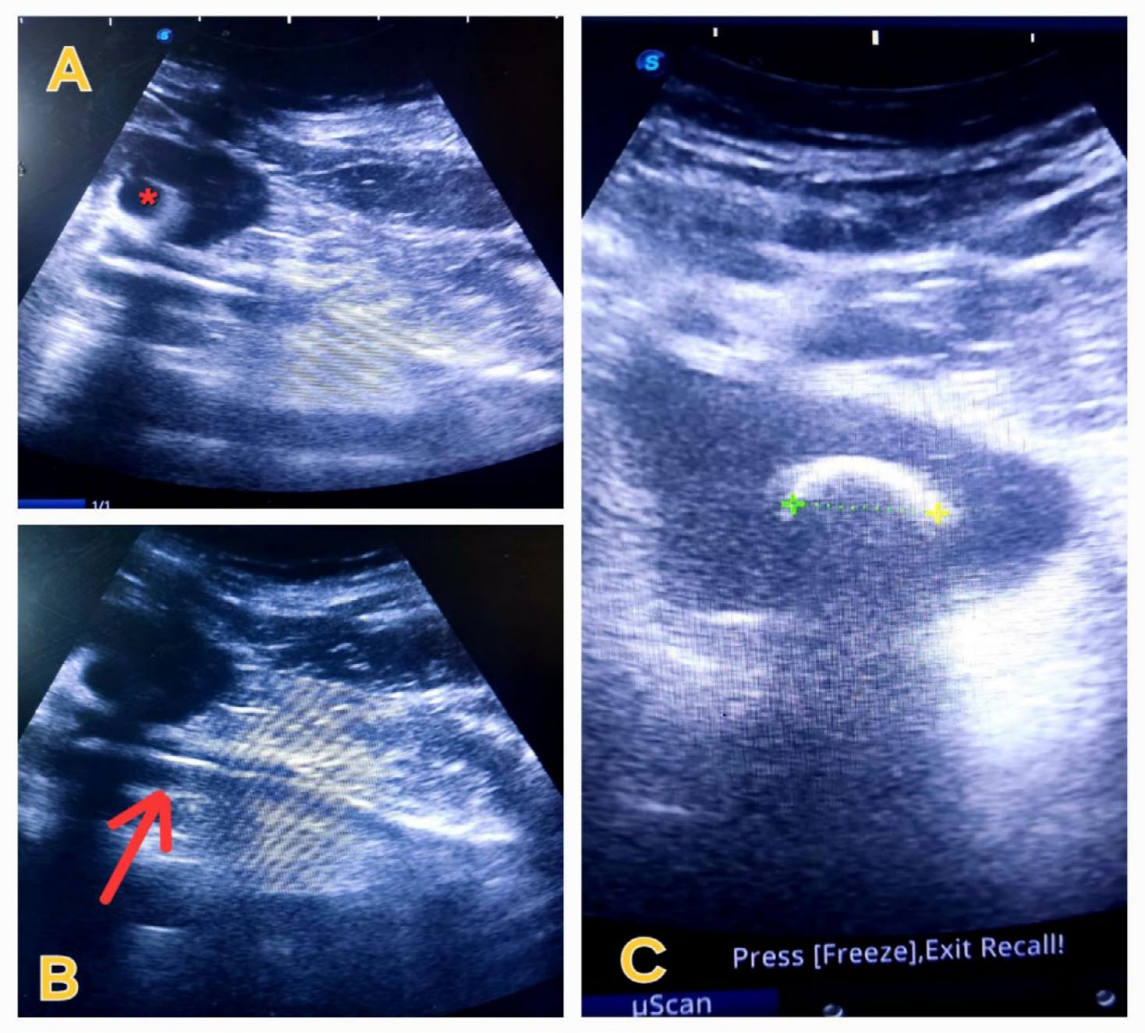
(A) Ultrasound scan showing right moderate hydronephrosis (red astric*). (B) Hyperechoic DJ stent in the right ureter (red arrow). (C) Encrusted lower end of DJ forming bladder stone.

**FIGURE 2:**
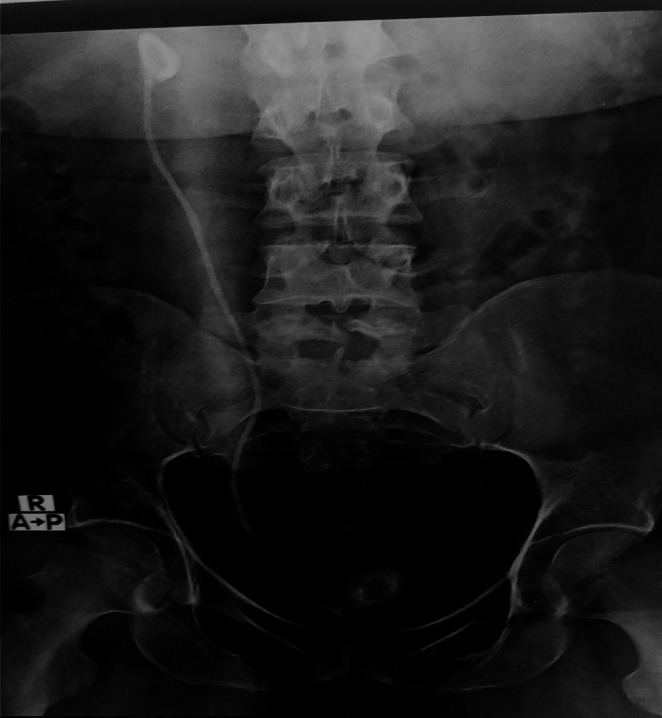
X‐ray ray KUB.

**FIGURE 3:**
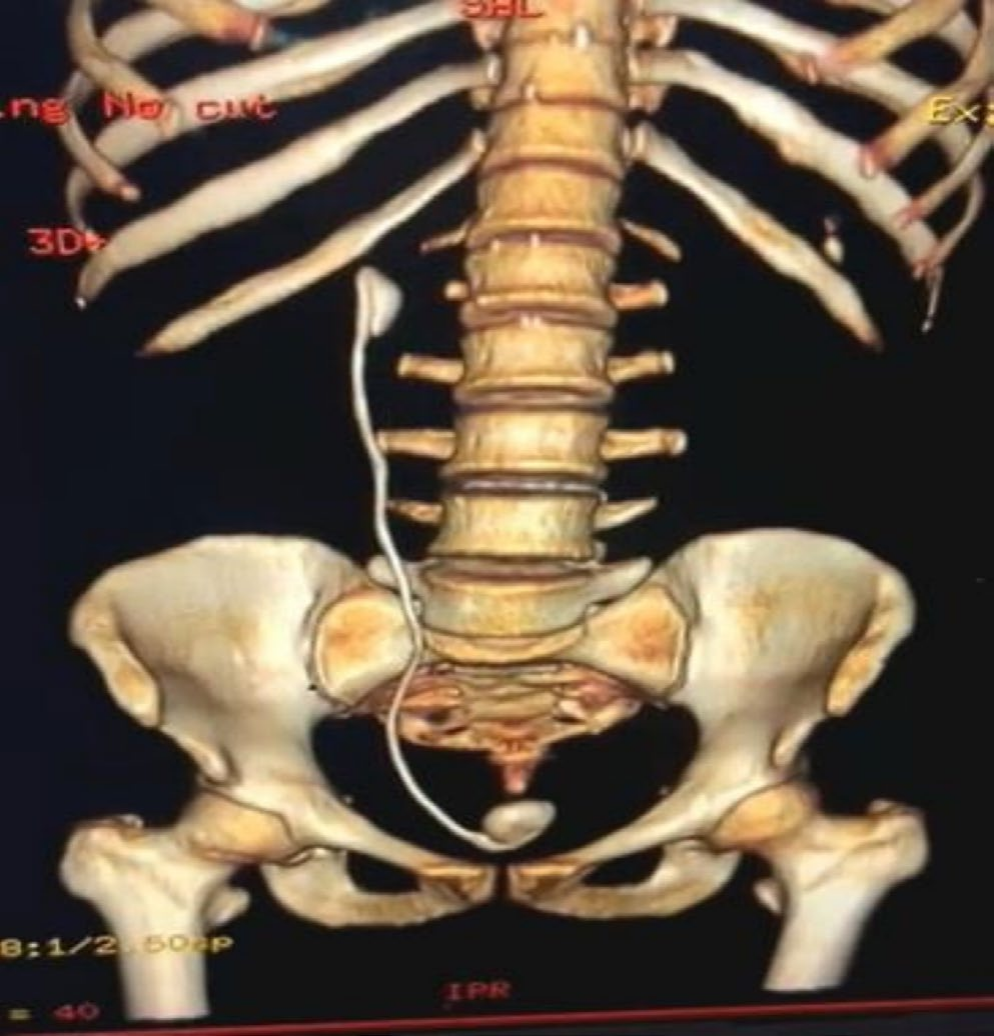
Computer tomography (CT) with 3D reconstruction.

Ultrasonographic (USG) assessment showed a hyperechoic structure in the bladder measuring a 23 mm calculus with posterior acoustic shadowing (Figure 1A–C). The size of the right kidney was normal but showed a hyperechoic structure with acoustic shadow, associated with moderate hydronephrosis (Figure 2A). The left kidney appeared sonologically normal. An X‐ray of the kidney, ureter, and bladder (KUB) showed an image of the double J stent in the right paravertebral space with two stones at the proximal and distal ends of the double J stent (Figure 2). A urological computed tomography (CT) scan showed a neglected double J stent in the right ureter with a renal stone attached to its proximal end with a size of 26 × 26 mm (density 1015 HU) and a bladder stone attached to its distal end with a size of 23 × 12 mm (density 812 HU) (Figure 3). Her hemoglobin was low at 7.9 mg/dL, urinalysis revealed uncountable pus cells, and urine culture showed growth of *Pseudomonas aeruginosa* spp. Renal function test was normal with s. creatinine 0.8 mg/dL and blood urea 16.

### 2.5 Diagnosis

The patient was diagnosed with a right neglected double‐J stent with renal and bladder stone formation.

### 2.6 Therapeutic Interventions

**FIGURE 4:**
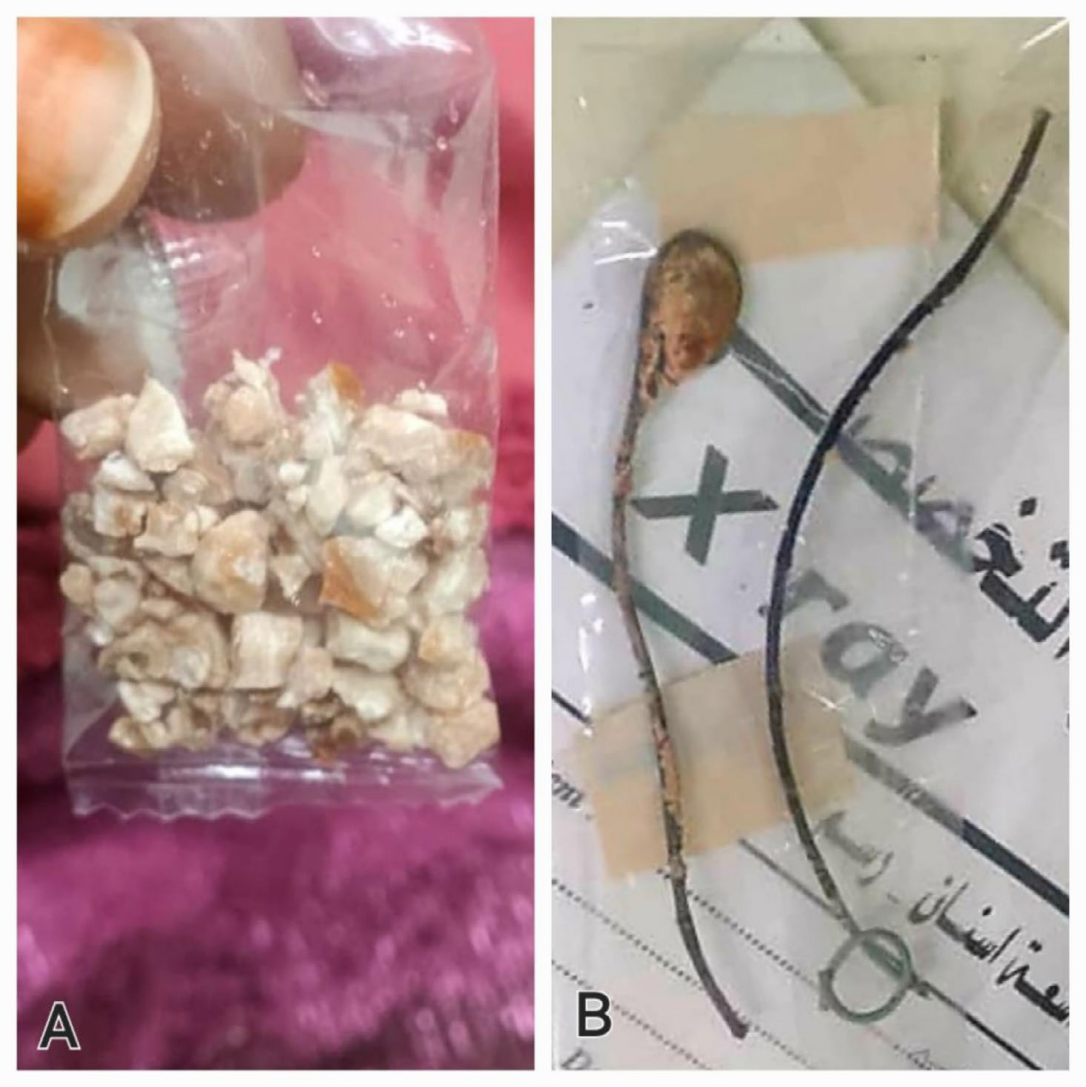
(A) Fragmented bladder stone. (B) Neglected DJ removed with stone.

Transurethral cystolitholapaxy was successfully performed to treat the bladder stone. However, the attempt to remove the DJ stent endoscopically was unsuccessful due to a large renal stone attached to the proximal end of the double J stent. As a result, the patient underwent pyelolithotomy to remove the stone from the renal pelvis via a flank incision, with the procedure carried out under general anesthesia (Figure 4).

### 2.7 Follow‐Up and Outcome of Interventions

During the postoperative evaluation 1 month later, the patient's overall condition was deemed satisfactory. She showed significant improvement, with no complications or issues observed, indicating a successful recovery process. The patient was in stable health and had returned to her usual level of well‐being.

## 3 Discussion

Since its introduction in 1967 by Zimskind et al., the DJ stent has become essential in urological procedures [1]. In the current case report, a DJ stent was inserted as a standard procedure following the ureteroscopic fragmentation of a ureteral stone. Typically, the DJ stent should be replaced or removed within 6 weeks to 6 months to prevent potential complications. However, if left in place too long or forgotten, it can cause significant complications, including infections, stone formation, and damage to the urinary tract. The primary risk factor for stent encrustation is the length of time the stent is in place. However, other contributing factors include metabolic or congenital abnormalities, stone disease, bacterial colonization, chemotherapy, pregnancy, and chronic kidney failure [5].

In the present case, the ureteral stent had been in place for a period of 4 years, which subsequently led to the formation of both a renal stone and a bladder stone. The prolonged duration of the stent in the urinary tract contributed to the development of these stones, highlighting the potential complications that can arise from long‐term stent retention. This situation underscores the importance of timely stent removal or replacement to prevent such adverse outcomes.

The longer a DJ stent remains in place, the higher the risk of stone formation. Rates of stent encrustation are reported as 9.2%, 47.5%, and 76.3% for stents left in place for 6 weeks, 6–12 weeks, and over 12 weeks, respectively [3].

Neglected double‐J stents have multiple contributing factors [1]. However, inadequate counseling and a lack of proper information provided to the patient and their family remain the primary causes, as highlighted by previous studies [6].

Another case reported that a giant bladder stone might occur due to neglected DJ stents. A lack of information and appropriate knowledge pertaining to the insertion of the ureteral stent is the main cause of bladder stone cases. Therefore, sharing information before and after the insertion of a ureteral stent and follow‐up of the health status of patients are important measures in managing the detrimental clinical effects of inserted ureteral stents in the urinary tract and kidney [7].

In the present case, the patient had limited information regarding the insertion of the ureteral stent during her procedure 4 years ago. As a result, she did not follow up regularly to monitor the condition of the stent. This case aligns with other studies involving neglected stent cases, highlighting the importance of proper patient education and routine monitoring to prevent complications. In our case, the patient has a history of stone disease, which is another risk factor.

The presentation of a neglected DJ stent can vary. In our case, the patient presented with flank pain. Similarly, in a study by Damiano et al., flank pain (25.3%) and storage lower urinary tract symptoms (18.8%) were the most commonly reported clinical presentations [8].

A study by Al‐Hajjaj et al. in Syria also reported intermittent right flank pain and irritative lower urinary tract symptoms [6].

Nir Tomer, Evan Garden, and their team developed a treatment plan combining the FECal and KUB systems. Encrustation over 5 mm should be assessed using KUB, CT, or ultrasound. Mild encrustation (< 5 mm and under 50% coverage) can be treated with cystoscopic removal. Severe encrustation (over 50% or 5 mm thick) requires surgery. For encrustations smaller than 1.5 cm, extracorporeal shock wave lithotripsy is preferred, while larger ones may require percutaneous nephrolithotomy [9].

Like our patient, stents with encrustation at both ends, including fragmentation, often require a multi‐step approach for removal. In this case, PCNL, flexible URS with holmium laser, and cystolitholapaxy were recommended. Due to resource limitations, a combination of endoscopic and open surgery was used with a successful outcome. We suggest this method as an effective alternative for healthcare settings in underdeveloped countries with limited resources.

### 3.1 Limitations

Management in this case was influenced by several limitations typical of resource‐limited settings, including the absence of a structured stent follow‐up system, limited patient education on stent care, and lack of access to advanced endourological tools. These constraints necessitated open surgery and underscored the need for context‐adapted strategies to prevent similar complications.

## 4 Conclusion

In this case report, the development of renal and bladder stones was likely a result of neglected DJ stents. Insufficient knowledge and understanding regarding the ureteral stent placement were key factors leading to these complications. Therefore, providing thorough information both prior to and following the insertion of a ureteral stent, along with regular follow‐up to monitor the patient's health, is an essential step in preventing the adverse effects associated with stent placement in the urinary system and kidneys.

## 5 Patient Perspective

The patient reported complete resolution of loin pain and expressed satisfaction with the treatment outcome, noting the absence of symptoms and the ability to resume normal daily activities without any limitations or discomfort.

## Author Contributions

**Abubaker Yassin:** conceptualization, data curation, investigation, software. **Osama Mohamed:** data curation, formal analysis, methodology, project administration, resources, supervision, validation, visualization, writing – original draft, writing – review and editing.

## Ethics Statement

This work was conducted according to the ethical guidelines of the Helsinki Declaration and was approved by the ethical committee of the hospital.

## Consent

The patient provided explicit written consent for the publication of their clinical information. All personal details have been thoroughly anonymized to safeguard privacy, and every effort has been made to ensure that the likelihood of identifying the patient is extremely low. This approach ensures that the information shared remains confidential while still contributing to the medical knowledge base.

## Conflicts of Interest

The authors declare no conflicts of interest.

## Data Availability

The data that support the findings of this study are available from the corresponding author upon reasonable request.
